# Large-Sample Genomic Data Mining for Quantitative Traits in U.S. Holstein Cows

**Published:** 2024-05-13

**Authors:** Yang Da, Dzianis Prakapenka, Zuoxiang Liang

**Affiliations:** Department of Animal Science, University of Minnesota, Saint Paul, USA

**Keywords:** Single nucleotide polymorphism, Genomic evaluation, Epistasis

## Abstract

The U.S. Holstein cattle have unprecedentedly large samples for genomic evaluation with genotypes of Single Nucleotide Polymorphism (SNP) markers and phenotypic observations of dairy quantitative traits. Such large samples provided unprecedented opportunities for the discovery of genetic variants and mechanisms affecting quantitative traits in Holstein cattle. Recent studies using the Holstein large samples on finding genetic variants affecting quantitative traits included a fat percentage study and two studies on reproductive traits. The fat percentage study confirmed that a chromosome region interacted with all chromosomes and the reproductive studies detected sharply negative homozygous recessive genotypes that were recommended for heifer culling. These novel findings provided examples showing the power of large-sample genomic mining for quantitative traits.

## ABOUT THE STUDY

**Figure 1:**
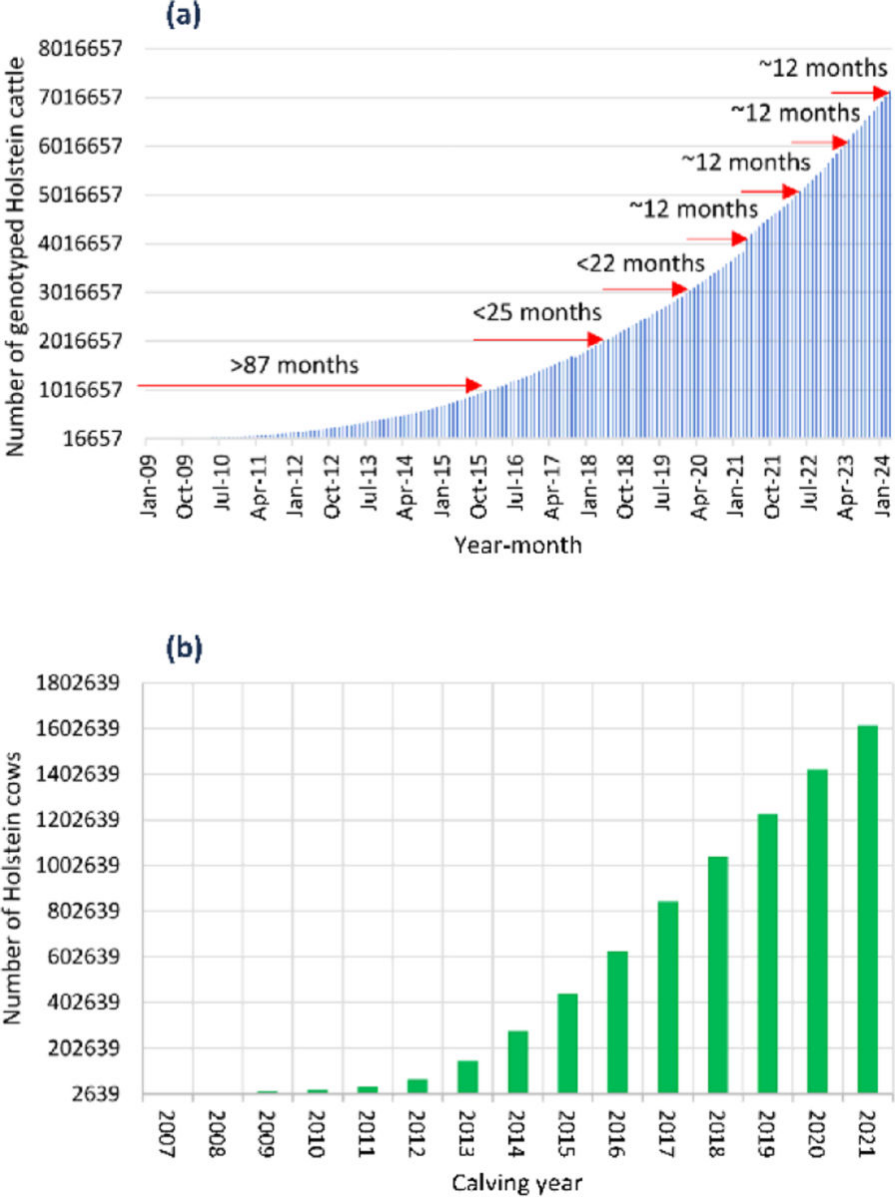
Growth of the genomic evaluation data of U.S. Holstein cattle. (a) Number of SNP genotyped Holstein cattle January 2009 through January 2024. (b) Number of SNP genotyped Holstein cows with phenotypic observations on milk yield in calving years 2007–2021 (61,764 cows calved in 2022 not shown due to incomplete calving year). Data source: Council on Dairy Cattle Breeding (CDCB).

The U.S. Holstein cattle have the largest samples of genomic evaluation data in domestic animals in the world. By March 2024, the U.S. Holstein breed had 7,147,052 SNP genotyped cattle with an increase about one million genotyped cattle a year since 2020 (Figure 1a). By the end of 2023, the number of genotyped Holstein cows with phenotypic observations exceeded 1.6 million with an annual increase of approximately 200,000 cows per year since 2016 (Figure 1b).

January 2009 through January 2024; (b) Number of SNP genotyped Holstein cows with phenotypic observations on milk yield in calving years 2007–2021 (61,764 cows calved in 2022 not shown due to incomplete calving year). Data source: Council on Dairy Cattle Breeding (CDCB).

Such large samples provided unprecedented statistical power for identifying genetic mechanism affecting quantitative using the approach of Genome-Wide Association Study (GWAS), particularly for detecting complex genetic mechanism that may include gene interaction effects commonly referred to as epistasis and multiple genetic factors with small effects. This is among the most difficult genetic areas due to difficulties to discover and the need of large samples to detect many small effects.

### The epistasis tests of A × A epistasis effects for fat percentage

The epistasis GWAS for fat percentage using 1,231,898 first lactation cows and 75,198 SNPs [1] had over 2.827 billion SNP pairs. For each SNP pair, four tests could be done, A×A, A×D, D×A and D×D epistasis effects, where ‘A’ stands for additive effect and ‘D’ stands for dominance effect. However, for computing feasibility and the purpose of validating interchromosome A × A epistasis effects with a chromosome14 region, only inter-chromosome SNP pairs between chromosome 14 and the remaining chromosomes (nearly 199 million pairs) were tested for A × A epistasis effects. Many of the allelic combinations of the SNP pairs had small effects and low frequencies, but the large sample detected 2763 pairs of significant inter-chromosome A × A effects with high statistical confidence from the nearly 199 million tests, confirming that one piece of chromosome 14 interacted with all chromosomes for fat percentage. This result was first discovered using 294,079 cows [2], and was the only such a result in any species. Given the uniqueness of this discovery, additional confirmation should be needed. The study using 1.2 million cows provided high-confidence confirmation of this unique discovery and in the meantime detected many new inter-chromosome A × A epistasis effects.

### Detection of rare but sharply negative recessive genotypes for reproductive traits

A Holstein GWAS on three fertility traits using over one million cows [3], and another GWAS on age at first calving using over 800,000 cows [4], also provided examples of the power of large-sample discovery of genetic factors for quantitative traits. These studies discovered some low-frequency but sharply negative recessive genotypes for the four reproduction traits and most of these recessive genotypes had not been detected using sample sizes of 186,188–269,158 Holstein cows that were already unprecedentedly large samples in 2019. The sharply negative recessive genotypes accounted for about 2% of Holstein cow population and were recommended for culling heifers carrying any of the recessive genotype so that farmers can avoid economic losses for raising those heifers to reproductive age to find the reproductive problems of those heifers.

### Computing challenges for genome-wide epistasis tests

The GWAS for inter-chromosome A × A between chromosome 14 and other chromosomes was a computing change whereas the GWAS for additive and dominance effects of the four reproductive traits was not a computing challenge for the Atlas computing system of USDA/ARS we used. The analysis was performed on Atlas ‘big memory’ partition with 1,546.595 GB of available RAM. For computing efficiency, intra-chromosome SNP pairs were skipped, and only inter-chromosome SNP pairs between chromosome 14 and the other 30 chromosomes were tested, where the pseudo-autosomal and nonrecombining regions of the X chromosome were treated as two chromosomes. Each pair of chromosomes was tested separately and used about 195.5 GB RAM and 3 hours to finish. All 30 runs took about 90 hours to finish. The Atlas system could only analyze 500,000 individuals for testing all 2.827 billion SNP pairs for A × A, A × D, D × A and D × D epistasis effects per SNP pair even when the ‘big memory’ partition was used. Since the Atlas system was shared by many users and the ‘big memory’ partition was not always available, pairwise epistasis testing was a computing challenge.

### The road “from phenotype to genotype and back from genotype to phenotype”

**Figure 2:**
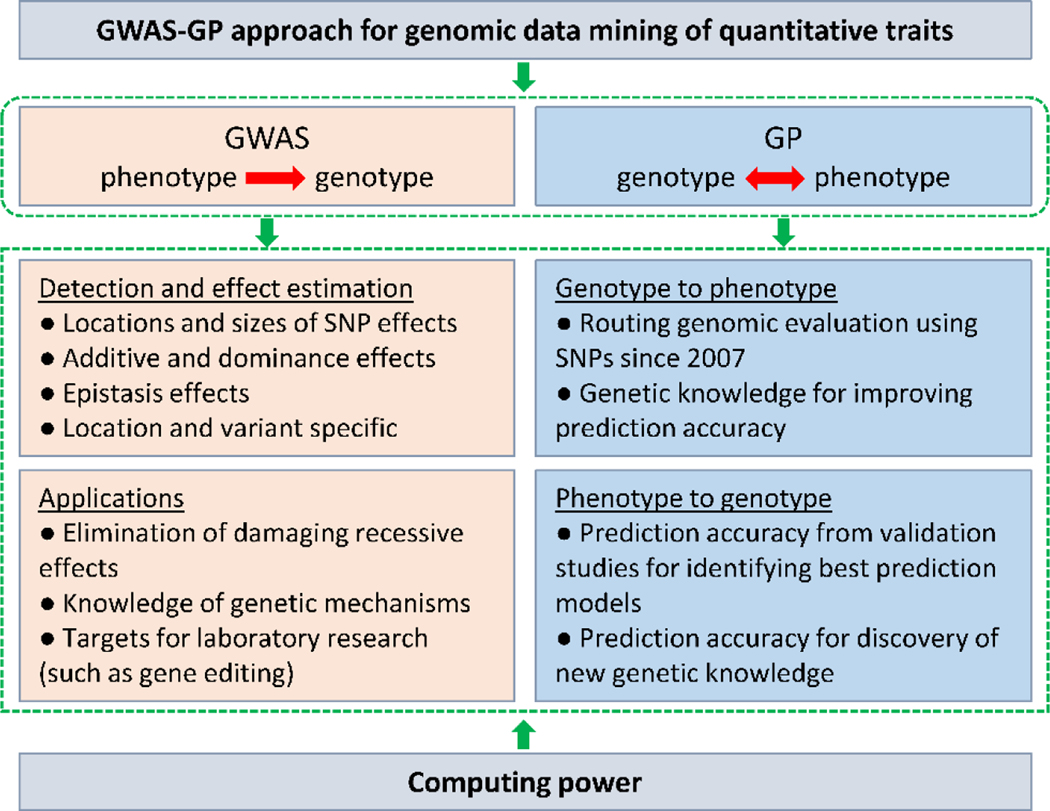
Genotype-phenotype round trip using the combined approach of GWAS and GP.

At the 2022 and 2023 NSF/NIH EDGE awardee meetings, a theme of discussion was “from phenotype to genotype and back from genotype to phenotype”, to be short named “genotype-phenotype round trip”. The combined approach of GWAS and Genomic Prediction (GP) using large Holstein samples provides a unique association-based non-laboratory approach for the genotype-phenotype round trip (Figure 2).

The GWAS approach can identify the locations and sizes of SNP effects as well as gene actions as shown by our examples of Holstein GWAS. Results of GWAS can be used for eliminating damaging recessive effects and provide targets for laboratory research such as gene editing. The GWAS approach investigates the path “from phenotype to genotype”.

The GP approach involves both paths “form genotype to phenotype” and “from phenotype to genotype”. GP predicts genetic and/or phenotypic values typically using genome-wide SNPs and has rapidly become a routine approach for genomic evaluation in many livestock and crop species since 2007.

An advantage of GP is the availability of an objective judgement for the performance of a prediction model through validation studies: the prediction model with the highest prediction accuracy is the best prediction model. Through validation studies, we have reported several examples of improved accuracy of GP using haplotype and epistasis effects in human, swine, and Holstein cows [5–7], as examples of the GP path “from genotype to phenotype”. The validation studies also generated interesting new genetic knowledge, providing examples of the GP path “from phenotype to genotype”. These examples included haplotypes of coding genes that improved the prediction accuracy [5,6], haplotypes of noncoding genes that were strikingly more accuracy than SNPs in those genes [5], and confounding between intra-chromosome A × A effects and additive effects [7]. In addition to interesting genetic knowledge from validation studies, the GP model can provide heritability estimates for any SNP, haplotype blocks or chromosome regions,providing genetic knowledge not commonly available from GWAS. For human High-Density Lipoproteins (HDL), the *CETP* gene was widely confirmed to have the most significant SNP effect by multiple human GWAS reports (https://www.ebi.ac.uk/gwas/). Our GP research showed also had*CETP* the highest SNP heritability and the highest haplotype heritability when each haplotype block was 50 Kb in size [6].

**Figure 3:**
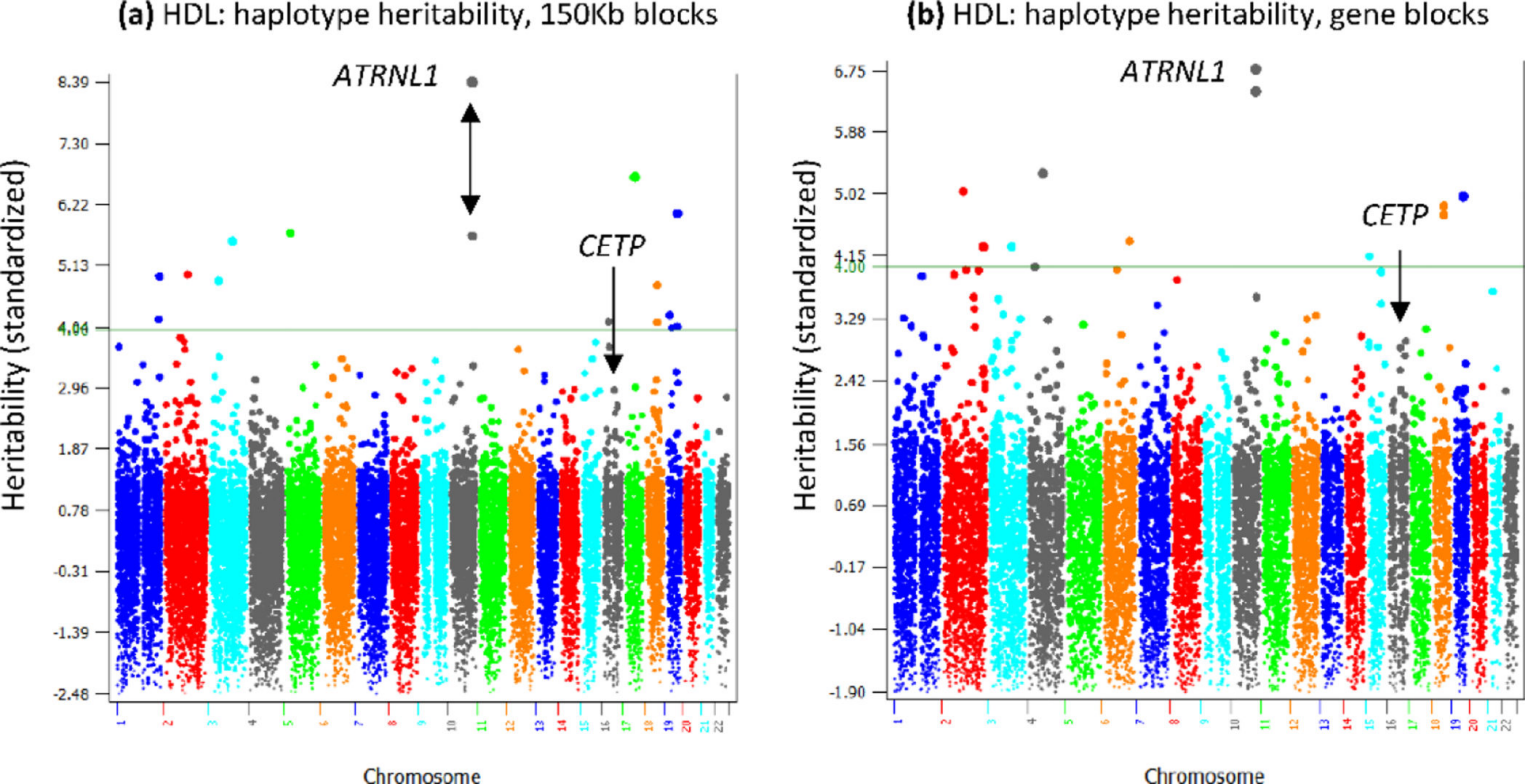
Genomic haplotype heritability estimates of two haplotype blocking methods for High Density Lipoproteins (HDL). a) The *ATRNL1* gene had the highest haplotype heritability for 150 Kb haplotype blocks; b) The *ATRNL1* gene had the highest haplotype heritability for gene-based haplotype blocks, where *ATRNL1* was divided into two blocks.

However, *CETP* no longer had the highest haplotype heritability when the size of the haplotype block increased to 150 Kb or when each gene was treated as a haplotype block. In these cases, the *ATRNL1* gene had the highest haplotype heritability, and many other genes also had higher haplotype heritability than that of *CETP* (Figure 3).

The GP example showed that GP can be a valuable approach for researching genotype-phenotype round trip”. The main challenge of the GP approach is the computing difficulty: GP can be far more demanding computationally than GWAS. Given computing power the GP approach using large samples is expected to identify effect types relevant to prediction accuracy and exclude effect types without contribution to prediction accuracy.

### A golden era for genomics data mining for quantitative traits in livestock species

The large-sample Holstein GWAS showed the unique opportunities for the discovery of genetic factors and mechanisms affecting quantitative traits. Such large samples could have been unimaginable only a decade ago. Although U.S. Holstein cattle have the largest sample sizes of genomic evaluation data, other livestock species with routing genomic evaluations including Jersey cattle, beef cattle, swine, and chicken are also accumulating genomic evaluation data and may soon have sample sizes comparable to the current Holstein sample sizes. The different species have many different quantitative traits and even the same trait of two different species may involve different genetic mechanisms. Many new discoveries of genetic factors and mechanisms affecting quantitative traits of those livestock species can be expected from large-sample analysis of the rapidly growing genomic evaluation data. The coming decade for livestock species with routine genomic evaluations is a golden era with unprecedented opportunities for the genetic research of quantitative traits.
